# Successful Management of Medication-Related Osteonecrosis of the Jaw (MRONJ) Surgically Treated by the Fragmentation Technique: A Case Report

**DOI:** 10.7759/cureus.110908

**Published:** 2026-06-15

**Authors:** Jean Martinien Engoue, Baraa Shamsi-Basha, Théo Bichet, Paul Juillard-Marchay, Alp Alantar

**Affiliations:** 1 Oral Surgery Unit, Hôpital Max Fourestier, Nanterre, FRA

**Keywords:** antibiotherapy, bone healing, bone sequestrum, dental extraction, fragmentation, medication-related osteonecrosis of the jaw (mronj)

## Abstract

An 82-year-old woman was referred with a post-extraction bone sequestration to the Unit of Oral Surgery. She was treated for osteoporosis with bisphosphonate (BP; alendronic acid, 70 mg/week) for 15 years. According to the clinical signs, a stage 2 medication-related osteonecrosis of the jaw (MRONJ) was diagnosed. Preoperative treatment consisted of antibiotherapy combined with 0.12% chlorhexidine mouthwash. A specific surgical approach was used, sequestrectomy by fragmentation of the bone sequestrum. Antibiotherapy was continued until the bone was completely covered by the gum. The clinical and radiological controls at 24 months and 30 months, respectively, were satisfactory. Excision of bone sequestra measuring more than 10 mm by the fragmentation technique could minimize operative trauma and thereby recurrence. This hypothesis will have to be confirmed by clinical studies.

## Introduction

The development and administration of bisphosphonates (BP) and denosumab have led oral surgeons to increasingly manage medication-related osteonecrosis of the jaw (MRONJ). Asymptomatic and non-extensive MRONJ is the most common type (95%) [1]. The hallmark feature of MRONJ is the area of exposed, necrotic, non-healing, asymptomatic bone for more than eight weeks. The mean age of onset of MRONJ is 66.5 ± 4.7 years, and it has a male-to-female ratio of 1:2. The mean duration of BP administration is 38.2 ± 15.7 months [2]. The triggering factor of MRONJ is tooth extraction, and its incidence varies between 0.3% and 9.0 % [3], with a 4.34% mean incidence [4]. Individuals may develop post-extraction MRONJ with 0.6% incidence when treated with oral bisphosphonates [5]. Although the excision of oral bone sequestra induced by bisphosphonate-related osteonecrosis of the jaw (MRONJ) is well documented, the fragmentation technique has been very rarely proposed. However, this original approach could minimize operative trauma and thereby recurrence. This hypothesis is illustrated by the present case report.

## Case presentation

An 82-year-old woman was referred with a bone sequestration (Figure 1) that had occurred after a recent extraction of the mandibular incisors. She was treated for osteoporosis with oral BP (alendronic acid, 70 mg/week) for 15 years. She had chronic respiratory failure with a history of thrombophlebitis and pulmonary embolism. She was treated by aerosols (budesonide/formoterol, tiotropium bromide, 2-agonist terbutaline, ipratropium, and antibodies (omalizumab)), furosemide 10 mg, and potassium chloride. Her surgical history revealed extractions of the incisors performed without antibiotic prophylaxis.

**Figure 1:**
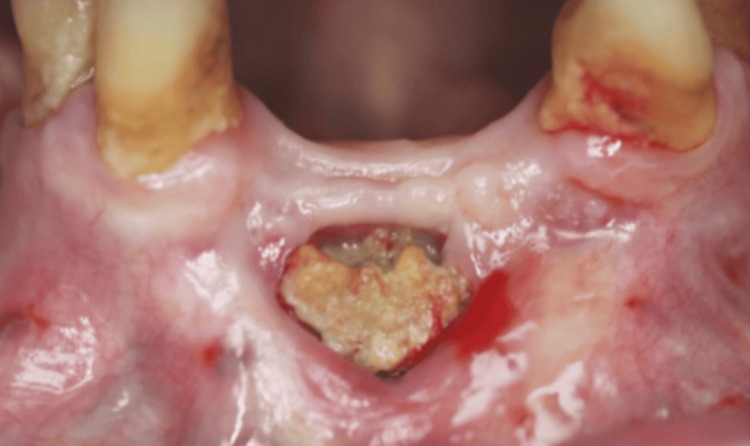
Extraction-related jaw osteonecrosis in the mandibular anterior sextant Extraction-related jaw osteonecrosis in a female treated with alendronic acid 70 mg/week for 15 years. Notice a 10 × 5 mm area of exposed, necrotic, asymptomatic bone. Image by Dr. A. Alantar

Exo-oral examination revealed submental lymph nodes; the endo-oral examination revealed a bone sequestrum of size 10 × 5 mm in the mandibular anterior sextant. The lesion was painless but annoying to the patient during chewing. The panoramic view (Figure 2) is not specific, as a bony sequestrum is a dense bone lesion.

**Figure 2:**
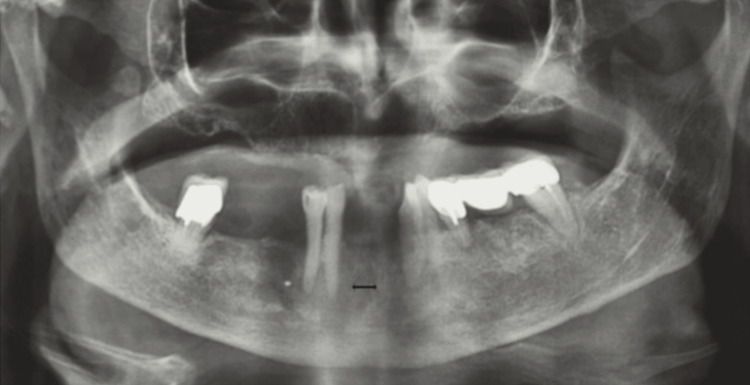
Panoramic view on the day of examination Notice the slight bony hypodensity (arrow). Distally to tooth #45, notice the asymptomatic cicatricial intraosseous radiolucency.

The diagnosis of stage 2 MRONJ has been made. Preoperative informed consent was given to the patient. Initial treatment consisted of antibiotherapy (amoxicillin/clavulanic acid, 2 g/day, 15 days), combined with 0.12% chlorhexidine mouthwashes (3 rinses/day, 30 days). Based on our experience, a specific surgical approach was used to prevent any recurrence: (1) sequestrectomy by fragmentation of the bone sequestrum using a 23 mm Zekrya carbide bur with irrigation (Figure 3 and Figure 4), (2) sharp edge smoothing, (3) bone cavity filling with a collagen hemostatic sponge (Figure 5), and (4) soft tissue suturing with 4-0 resorbable Monocryl and simple interrupted pattern (Figure 6).

**Figure 3:**
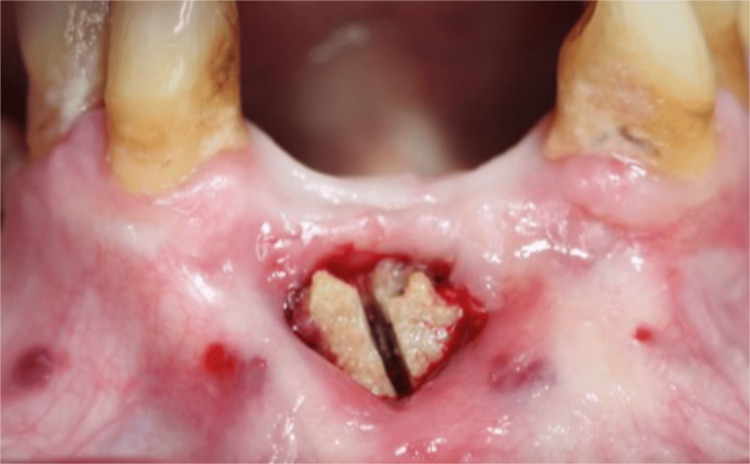
Fragmentation of the bony sequestrum Fragmentation into two pieces of the bony sequestrum with a Zekrya carbide bur.

**Figure 4:**
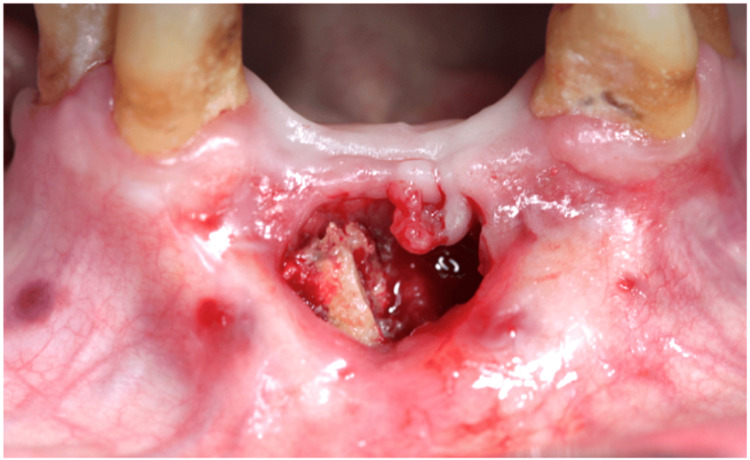
Fragmentation into two pieces of the bony sequestrum with a Zekrya carbide bur Partial resection of the bony sequestrum.

**Figure 5:**
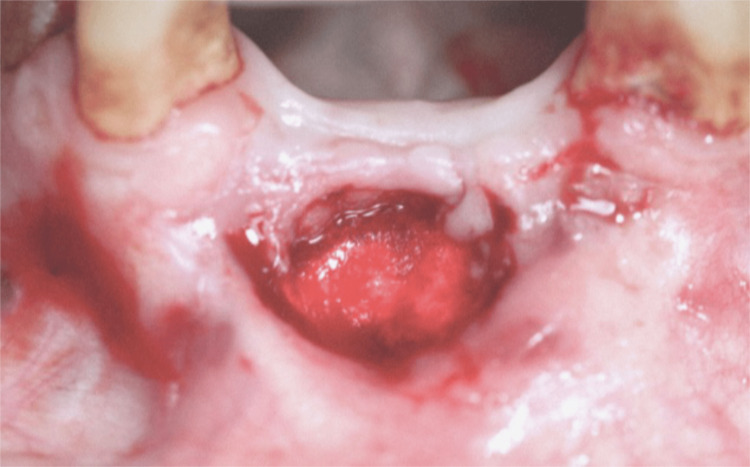
Hemostatic sponge Bone cavity filled with a collagen hemostatic sponge.

**Figure 6:**
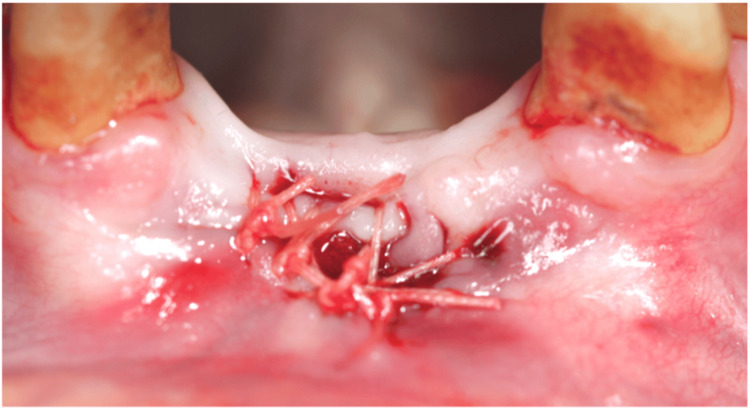
Suture view Soft tissue sutured with 4-0 resorbable Monocryl® and simple interrupted pattern. The sutures were placed without tissue tension.

No flap was raised. Antibiotherapy was maintained for 15 days until the bone was completely covered by the mucosa. Antimicrobial treatment was combined with chlorhexidine mouthwash (0.12%). Sutures were removed after 15 days. A partial denture with occlusal cleats (to avoid compression of the gingiva) was fabricated four months postoperatively. The clinical follow-up at 24 months (Figure 7) was satisfactory with an asymptomatic bone concavity, which is characteristic of bone healing in patients under treatment with BP. The radiological view was not specific. A biannual follow-up was instituted.

**Figure 7:**
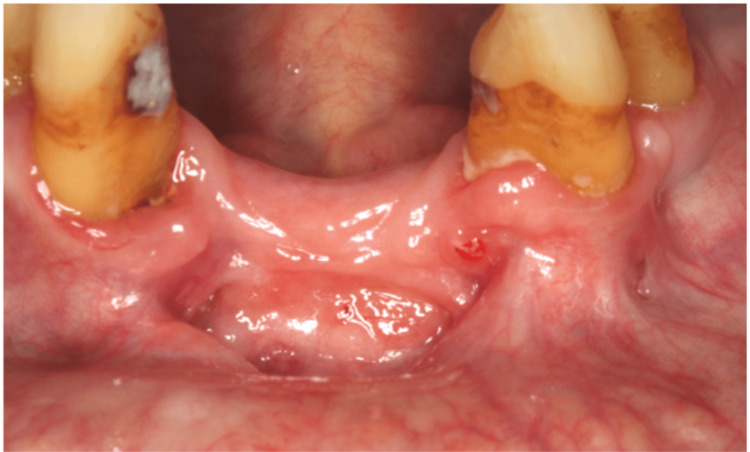
Clinical view at 24 months follow-up Notice the bony concavity, which is characteristic of bone healing in patients under treatment with BP. BP: bisphosphonate

## Discussion

The standard treatment for stage 2 MRONJ is a minimally invasive surgery by sequestrectomy, preserving the soft tissues, combined with local and general anti-infective treatments [6]. According to Blus et al., healing of treated sites with ultrasonic piezoelectric bone surgery may result from the synergic effect of bone ablation, biofilm alteration, and antibiotic administration [7]. This alteration of the biofilm may permit better access of antibiotics to the involved germs.

Regarding the surgical treatment of MRONJ, available evidence is insufficient to either claim or refute a benefit of association to low-level laser therapy (LLLT), plasma rich in growth factor (PRGF), cell therapy [8], hyperbaric oxygen therapy, tetracycline fluorescence-guided sequestrectomy [6,7], or pentoxifylline-tocopherol with clodronate therapy [9]. In extensive forms of stage 3 MRONJ, after a previous cone beam computed tomography (CBCT) exploration, flap coverage is required [10]. Early detection and follow-up of MRONJ with 99mTc scintigraphy has been proposed by Tanabe et al. [11]. As BPs were given for osteoporosis, no histopathological examination was needed in order to rule out any metastatic disease. To our knowledge, the fragmentation sequestration technique has not been explicitly described.

The management of MRONJ depends on the extent of necrosis, the underlying disease, the symptomatology, the administration route, and the cumulative dose of the implicated bone antiresorptive treatment [12]. The average healing time after sequestrectomy is three months for stage 2 and two months for stage 1 [13]. The success rate is 96.6% for low cumulative dose treatments and 51.5% for other doses. Giudice et al. recorded that surgical treatment of patients in the early stages of MRONJ guarantees benefits in outcomes such as mucosal integrity and lesion downstaging, and improvement in quality of life [13].

## Conclusions

Although the prognosis of stage 2 MRONJ is generally favorable, the excision of bony sequestrum measuring more than 10 mm could be optimized by fragmenting, thereby minimizing operative trauma and eventual recurrence. Patients must be warned that a bony cavity will form after healing. The effectiveness of bony sequestrum removal using the fragmentation technique should be confirmed by clinical trials.
